# Selective inhibition of somatostatin-positive dentate hilar interneurons induces age-related cellular changes and cognitive dysfunction

**DOI:** 10.1093/pnasnexus/pgad134

**Published:** 2023-04-13

**Authors:** Jinrui Lyu, Rajasekar Nagarajan, Maltesh Kambali, Muxiao Wang, Uwe Rudolph

**Affiliations:** Department of Comparative Biosciences, College of Veterinary Medicine, University of Illinois at Urbana-Champaign, Urbana, IL 61802-6178, USA; Neuroscience Program, College of Liberal Arts and Sciences, University of Illinois at Urbana-Champaign, Urbana, IL 61802-6178, USA; Department of Comparative Biosciences, College of Veterinary Medicine, University of Illinois at Urbana-Champaign, Urbana, IL 61802-6178, USA; Department of Comparative Biosciences, College of Veterinary Medicine, University of Illinois at Urbana-Champaign, Urbana, IL 61802-6178, USA; Department of Comparative Biosciences, College of Veterinary Medicine, University of Illinois at Urbana-Champaign, Urbana, IL 61802-6178, USA; Neuroscience Program, College of Liberal Arts and Sciences, University of Illinois at Urbana-Champaign, Urbana, IL 61802-6178, USA; Department of Comparative Biosciences, College of Veterinary Medicine, University of Illinois at Urbana-Champaign, Urbana, IL 61802-6178, USA; Carl R. Woese Institute of Genomic Biology, University of Illinois at Urbana-Champaign, Urbana, IL 61801, USA

**Keywords:** dentate gyrus, somatostatin, interneurons, cognition, chemogenetics

## Abstract

The cellular basis of age-related impairments of hippocampal function is not fully understood. In order to evaluate the role of somatostatin-positive (Sst^+^) interneurons in the dentate gyrus (DG) hilus in this process, we chemogenetically inhibited Sst^+^ interneurons in the DG hilus. Chronic chemogenetic inhibition (CCI) of these neurons resulted in increased c-Fos staining in the DG hilus, a decrease in the percentage of GAD67- and of Sst-expressing interneurons in the DG, and increased microglial activation in DG, CA3, and CA1. Total dendritic length and spine density were reduced in DG and CA1, suggesting reduced dendritic complexity. Behaviorally, the recognition index in an object recognition task and the percentage of spontaneous alternations in the Y-maze were decreased, while in both initial and reversal learning in the Morris water maze, the latencies to find the hidden platform were increased, suggesting cognitive dysfunction. Our findings establish a causal role for a reduced function of Sst^+^ interneurons in the DG hilus for cognitive decline and suggest that this reduced function may contribute to age-related impairments of learning and memory. Furthermore, our CCI mice may represent a cellularly defined model of hippocampal aging.

**Significance Statement**

Neuronal circuits and cellular processes underlying age-related cognitive dysfunction are not well understood. We observed that chronic chemogenetic inhibition of a defined cell type, somatostatin-positive (Sst^+^) interneurons in the dentate gyrus (DG) hilus, which have previously been found to be associated with cognitive dysfunction in aged rodents, is necessary and sufficient to elicit changes in the expression of interneuronal markers, an increase in the activity of DG granule cells, increased microglial activation across the entire hippocampus, and an impairment of learning and memory-related tasks. Thus, inhibition of Sst^+^ interneurons in the DG hilus replicates changes that are also seen with normal aging, representing a novel cellularly defined animal model of hippocampal aging.

## Introduction

Aging is an inevitable, complex, and multifactorial process. Cognitive decline, such as memory loss, is one of the most prevalent concerns in the aging population. The hippocampus plays a vital role in cognition, learning, and memory in the mammalian brain (1–3). Importantly, the number of GABAergic interneurons in the hippocampus is reduced by normal aging (4–6), and hilar interneuron vulnerability has been shown to be correlated with age-regulated memory impairment (6). Optogenetic inhibition of hilar GABAergic interneuron activity impairs spatial memory (7). Moreover, many studies showed that aging in rodents is associated with dentate gyrus (DG) and CA3 hyperactivity (8, 9), CA1 hypoactivity (9–11), and reduced glutamatergic and GABAergic signaling in the hippocampus (12).

The dentate gyrus (DG), the first input-receiving region in the trisynaptic hippocampal pathway, is located between the entorhinal cortex and the CA3 region of the hippocampus. It receives information from the entorhinal cortex and projects to the CA3 pyramidal neurons, which then project to CA1 pyramidal neurons. DG granule cells encode spatial and contextual information (13). The tonic inhibition of DG granule cells, which are main regulators of hippocampal neuronal activity, controls pattern separation by distinguishing overlapping interferences into distinct and nonoverlapping information (14). α5 subunit-containing GABA_A_ receptors (α5-GABA_A_R), which are strongly expressed in the hippocampus, where they are primarily located extrasynaptically, mediate tonic inhibition in DG granule cells, CA1 and CA3 pyramidal cells (14, 15). Mice with a conditional knockout of α5-GABA_A_R in DG granule cells, which display a reduced tonic inhibition but preserved phasic inhibition, perform worse in many behavioral tasks associated with high memory interference. These mice displayed increased c-Fos staining in DG and CA3, consistent with hyperactivity (14). Interestingly, hyperactivity of DG and CA3 has also been reported to be linked to age-related memory decline in aging humans (16).

Somatostatin (Sst)-expressing interneurons are a subpopulation of GABAergic interneurons. Somatostatin-positive (Sst^+^) interneurons regulate hippocampal networks through dendritic inhibition of the DG circuitry (17). In the ApoE4 KI model of Alzheimer's disease that showed learning and memory deficits, the numbers of hilar Sst^+^ interneurons were decreased age-dependently (18). After receiving compounds that reduced hippocampal activity, both aged rats and human patients with amnestic mild cognitive impairment improved their memory (19, 20). Moreover, age-related cognitive deficits have been found to be correlated with a decrease in Sst^+^ interneurons in the DG hilus in rats (6); however, a causal relationship has not been demonstrated. There are two functionally contrasting types of Sst^+^ cells in the DG hilus: hilar perforant path-associated interneurons targeting granule cells and providing dendritic inhibition to the DG circuitry and hilar interneurons providing perisomatic inhibition onto GABAergic cells in the DG and projecting to the medial septum (17).

In this study, we examined whether suppression of the activity of hilar Sst^+^ interneurons is sufficient to induce defined learning and memory deficits, and whether this would occur only with chronic or also with subchronic inhibition. Our primary hypothesis was that only chronic inhibition of Sst^+^ interneurons results in learning and memory deficits. We tested this hypothesis by generating and analyzing mice in which these neurons could be chemogenetically silenced by clozapine. If reduction of activity of Sst^+^ interneurons in the dentate hilus resulted in cognitive dysfunction, the mice with such changes would potentially also represent a novel mouse model of age-related cognitive decline.

## Results

**Fig. 1.**
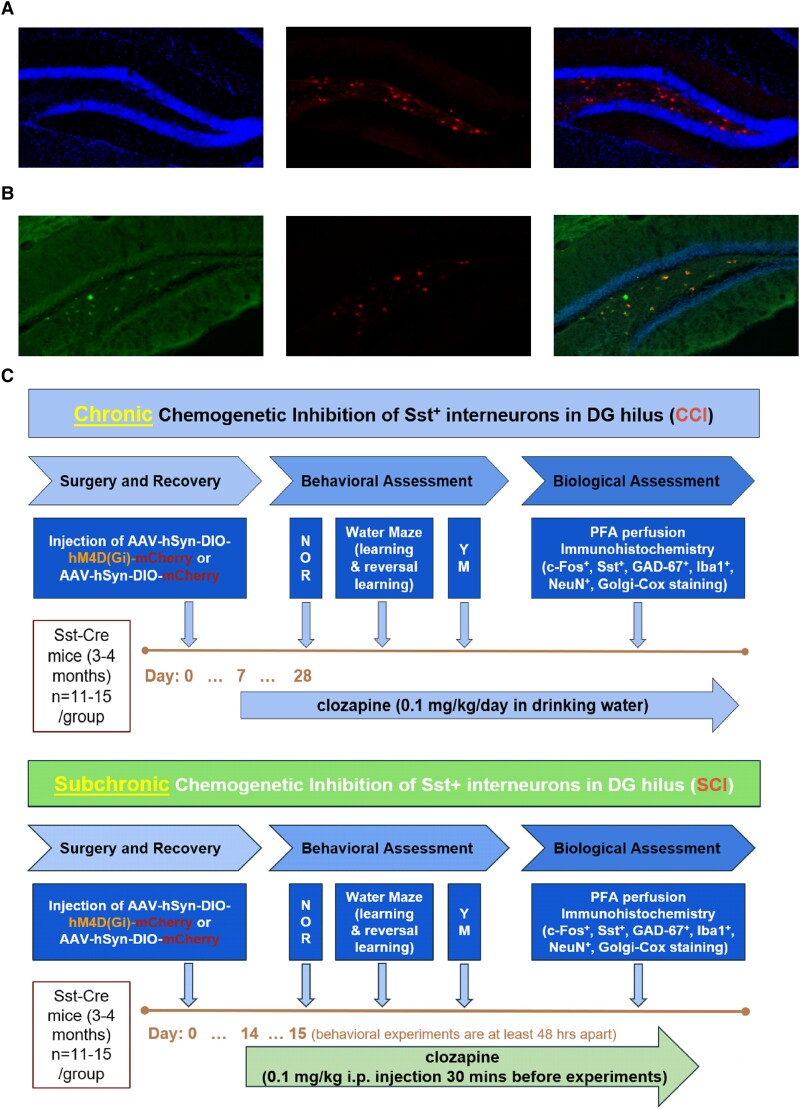
Experimental design of DG hilus Sst^+^ cell manipulation. A) Immunofluorescence staining of coronal sections from an AAV-hSyn-DIO-mCherry mouse showing DAPI counterstain (left: DAPI; middle: mCherry expression; right: merged image). B) Immunofluorescence colabeling of coronal sections from an AAV-hSyn-DIO-mCherry mouse (left: Sst labeling with Alexa Fluor 488; middle: mCherry; right: merged image, with DAPI). C) Top: chronic chemogenetic inhibition of DG hilus Sst^+^ cells. Bottom: subchronic chemogenetic inhibition of DG hilus Sst^+^ cells.

The goal of this study was to determine whether a factor that has been observed in aged animals, i.e. a decline in the number of Sst^+^ interneurons in the DG hilus, is sufficient to induce cognitive decline and may thus underlie age-related cognitive dysfunction. Aging being a chronic process, we compared the effects of chronic and subchronic chemogenetic inhibition (CCI and SCI, respectively) of these neurons at the cellular and behavioral levels in order to determine whether chronic inhibition is required for these changes (Fig. 1A and C). With a transduction efficiency of ∼63%, we are effectively looking at a partial inhibition of the activity of DG hilar Sst^+^ interneuron population (Fig. 1B).

### Cellular activity, interneuronal markers, and microglial activation

In order to assess how a reduced activity of hilar Sst^+^ interneurons affects the activity of principal neurons in hippocampal subregions, we performed c-Fos staining in DG hilus, DG granule cell layer (GCL), CA3, and CA1 subregions of the hippocampus.

**Fig. 2.**
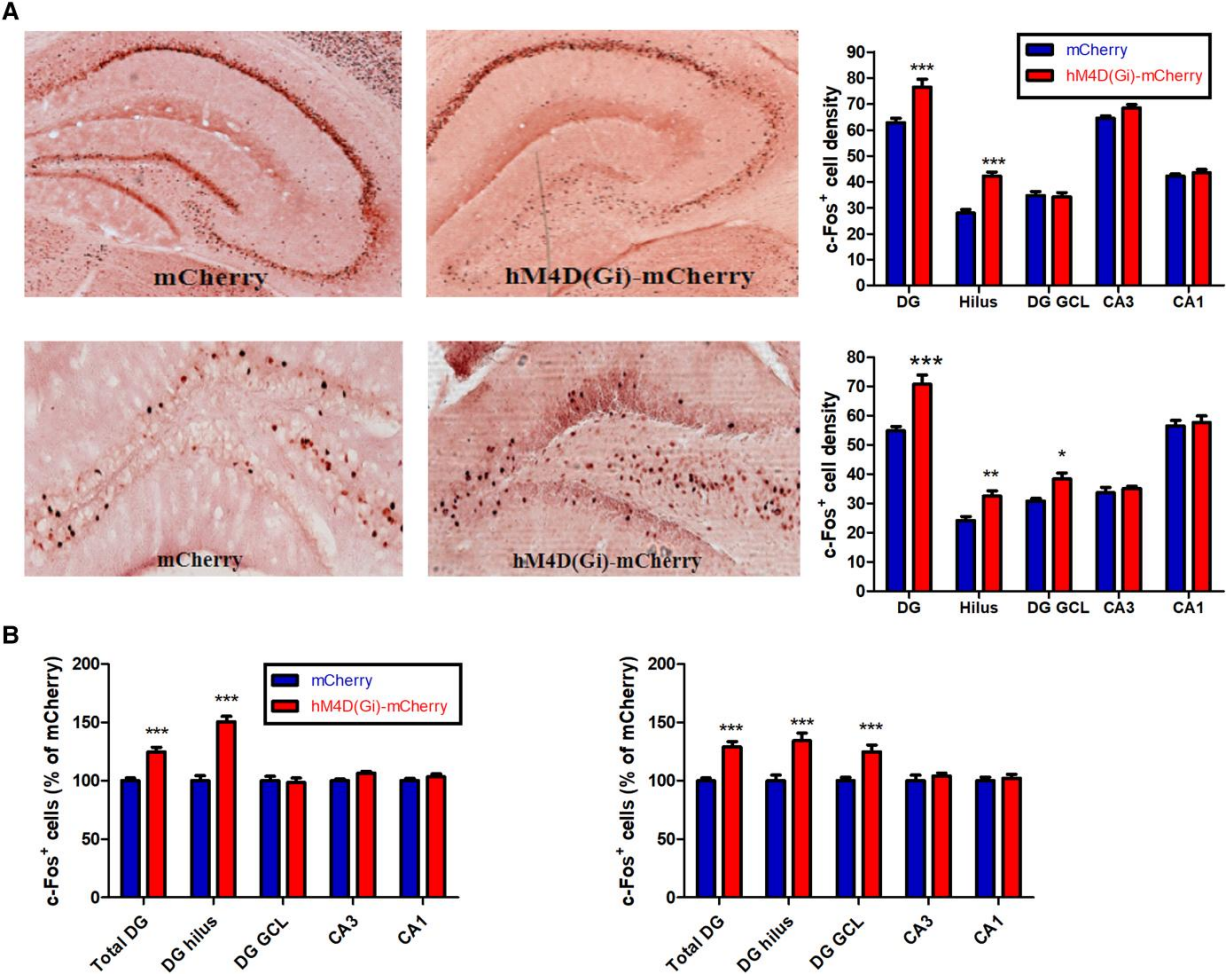
c-Fos^+^ cell counts in hippocampal subfields [total DG, DG hilus, DG granule cell layer (DG GCL), CA3, and CA1]. A) Top: representative sections showing c-Fos expression in AAV-hSyn-DIO-mCherry mice (“mCherry” mice) and AAV-hSyn-DIO-hM4D(Gi)-mCherry mice (“hM4D(Gi)-mCherry” mice) during CCI. Bottom: representative sections showing c-Fos expression in mCherry and hM4D(Gi)-mCherry mice during SCI. B) Left: estimated density of c-Fos^+^ cells in the hM4D(Gi)-mCherry mice after CCI, normalized to the number of c-Fos^+^ cells in the mCherry control mice. Right: estimated density of c-Fos^+^ cells in the hM4D(Gi)-mCherry mice after SCI, normalized to the number of c-Fos^+^ cells in the mCherry control mice. *P < 0.05, **P < 0.01, and ***P < 0.001 compared with the corresponding mCherry control group.

We observed that in mice with a CCI of Sst^+^ interneurons in the DG hilus, c-Fos staining in total DG (Diff = 13.65, *t* = 5.977) and in DG hilus (Diff = 14.22, *t* = 6.225) was increased (*P* < 0.001, *F*_(4,50)_ = 224.43, two-way ANOVA), consistent with hyperactivity; however, c-Fos staining in CA3 and CA1 was unaltered (*P* > 0.05, Fig. 2A, top panel). In mice with a SCI of Sst^+^ in the DG hilus, c-Fos staining also increased in DG hilus (Diff = 8.273, *t* = 3.096) and DG GCL (Diff = 7.584, *t* = 2.838) (*P* < 0.05, *F*_(4,50)_ = 133.77, two-way ANOVA), but not in CA3 and CA1 (*P* > 0.05, Fig. 2A, bottom panel). This suggests that both chronic and subchronic DG hilus-selective inactivation of Sst^+^ interneurons lead to increased c-Fos^+^ expression in the DG, possibly due to reduced tonic inhibition due to lack of an inhibitory input. When results were normalized to the number of c-Fos^+^ cells in mice infected with the control virus, CCI manipulation resulted in a 24.6% increase of c-Fos staining in the total DG (*P* < 0.001, *F*_(4,60)_ = 21.82, Diff = 24.6, *t* = 5.347, two-way ANOVA) with a 51% increase in the DG hilus (*P* < 0.001, Diff = 50.82, *t* = 4.928) (Fig. 2B, left panel) while SCI manipulation resulted a 29% increase in the total DG (*P* < 0.001, *F*_(4,60)_ = 5.967, Diff = 28.87, *t* = 4.779) with a 34% increase in the DG hilus (*P* < 0.001, Diff = 34.21, *t* = 5.664) and a 24% increase in the DG GCL (*P* < 0.001, Diff = 24.66, *t* = 4.083), which is an indication of hyperactivity in the DG region (Fig. 2B, right panel).

**Fig. 3.**
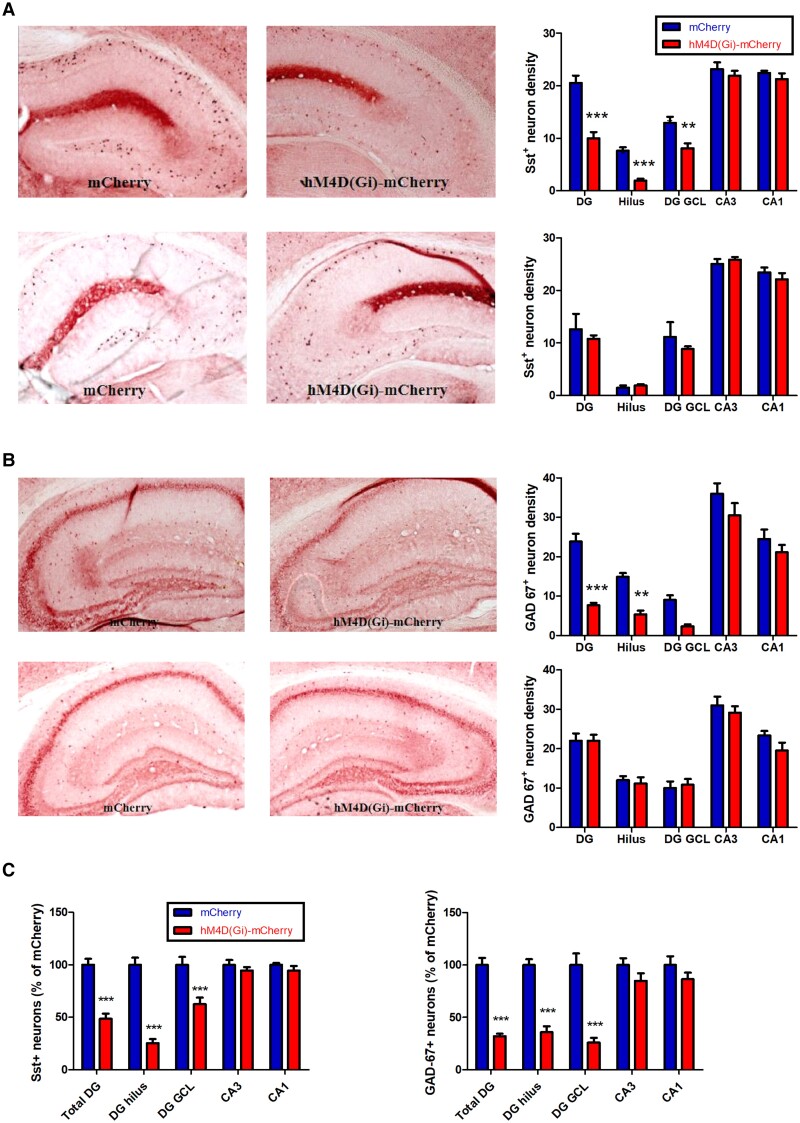
Sst^+^ neuron and GAD67^+^ neuron counts in hippocampal subfields. A) Top: representative sections showing Sst expression in AAV-hSyn-DIO-mCherry and AAV-hSyn-DIO-hM4D(Gi)-mCherry mice after CCI. Bottom: Sst expression after SCI. B) Top: representative sections showing GAD67 expression in AAV-hSyn-DIO-mCherry and AAV-hSyn-DIO-hM4D(Gi)-mCherry mice after CCI. Bottom: GAD-67 expression after SCI. C) Left: estimated density of Sst^+^ cells in hM4D(Gi)-mCherry mice after CCI, normalized to the number of Sst^+^ cells in the mCherry control mice. Right: estimated density of GAD-67^+^ cells in hM4D(Gi)-mCherry mice after CCI, normalized to the number of GAD-67^+^ cells in the mCherry control mice. **P < 0.01 and ***P < 0.001 compared with the corresponding mCherry control group.

We then assessed the influence of CCI and SCI of Sst^+^ interneurons on the interneuronal markers Sst and GAD67. While CCI mice displayed a significant decrease of Sst staining in DG (total DG: *P* < 0.001, *F*_(4,50)_ = 114.1, Diff = −10.53, *t* = 7.43; hilus: *P* < 0.001, Diff = −5.694, *t* = 4.023; GCL: *P* < 0.001, Diff = −4.833, *t* = 3.415) (Fig. 3A, top panel), GAD67^+^ was significantly decreased only in DG hilus (*P* < 0.01, *F*_(4,50)_ = 70.36, Diff = −9.540, *t* = 3.675) (Fig. 3B, top panel). In SCI mice, no significant change was observed with both Sst and GAD67 stainings (*P* > 0.05, two-way ANOVA) (Fig. 3A and B, bottom panels). Compared with mice infected with the control vector, we observed a 51% reduced density of Sst^+^ interneurons in the total DG region (*P* < 0.001, *F*_(4,60)_ = 16.72, Diff = −51.26, *t* = 6.985, two-way ANOVA) with a 75% reduction in the DG hilus (*P* < 0.001, Diff = −74.65, *t* = 10.17) and a 37% reduction in the DG GCL (*P* < 0.001, Diff = −37.44, *t* = 5.102) with the CCI treatment (Fig. 3C, left panel). For GAD67^+^ neurons, there is a 68% reduction in density in the total DG region (*P* < 0.001, *F*_(4,60)_ = 9.717, Diff = −67.89, *t* = 7.069, two-way ANOVA) with a 64% reduction in density in the DG hilus (*P* < 0.001, Diff = −64.14, *t* = 6.679) and 74% reduction in density in the DG GCL (*P* < 0.001, Diff = −74.07, *t* = 7.713) (Fig. 3C, right panel).

**Fig. 4.**
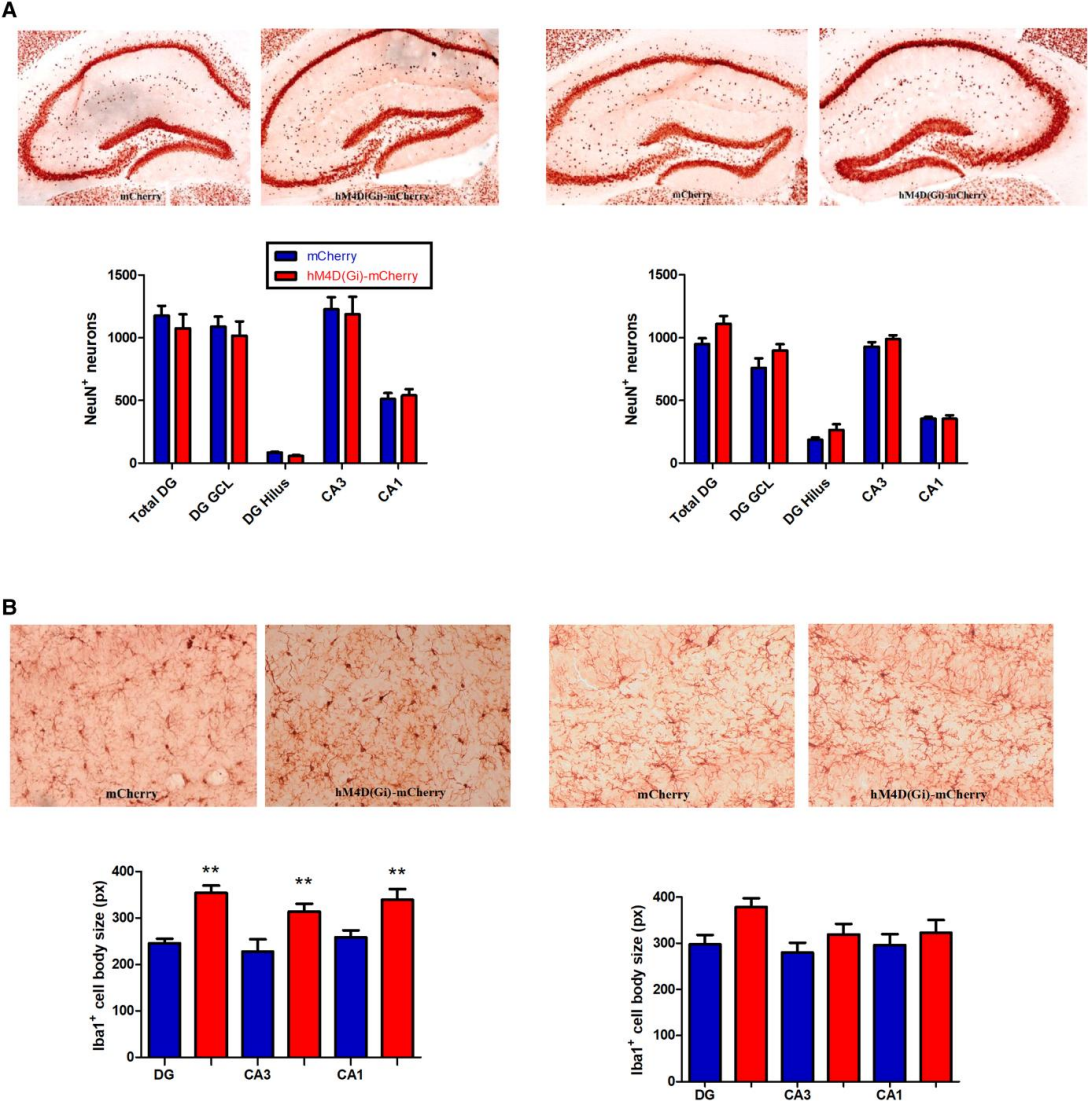
NeuN^+^ neuron counts and Iba1^+^ cell body size in hippocampal subfields. A) Left: representative sections showing NeuN expression in AAV-hSyn-DIO-mCherry and AAV-hSyn-DIO-hM4D(Gi)-mCherry mice after CCI. Right: representative sections showing NeuN expression in mCherry and hM4D(Gi)-mCherry mice after SCI. B) Left: representative sections showing Iba1-immunostained areas with pixels (px) indicating cell body size in mCherry and hM4D(Gi)-mCherry mice after CCI. Right: representative sections showing Iba1-immunostained areas with px indicating cell body size in mCherry and hM4D(Gi)-mCherry mice after SCI. **P < 0.01 compared with the corresponding AAV-mCherry group.

We performed staining of Iba1 (*I*onized Ca^2+^-*b*inding *a*dapter protein 1), a marker constitutively expressed by microglia, in brain sections, as increased Iba1 expression could serve as a proxy for microglial activation, allowing us to investigate whether chemogenetic inhibition led to hippocampal microglial activation. Whereas SCI did not result in changes in in an increase in Iba1^+^ cell body size (Fig. 4B, right panel, *P* > 0.05), CCI resulted in an increased Iba1^+^ cell body size in DG (*q* = 5.821, *P* < 0.001, one-way ANOVA), CA3 (*q* = 4.757, *P* < 0.01), and CA1 (*q* = 4.480, *P* < 0.01) (Fig. 4B, left panel). As a control experiment, we stained for NeuN^+^, which is a broadly expressed neuronal marker. Neither SCI (*F*_(4,25)_ = 0.8259, *P* > 0.05, two-way ANOVA) nor CCI (*F*_(4,25)_ = 0.1460, *P* > 0.05, two-way ANOVA) resulted in a change in the number of NeuN^+^ neurons (Fig. 4A). These data suggest a reduced expression of the markers Sst and GAD67 in DG hilar interneurons and in the DG GCL in the absence of a loss of neurons, and that this change results in increased hippocampal microglial activation in DG, CA3, and CA1. As it has been reported recently that microglia is involved in negative feedback control of neuronal activity (21), this microglial activation might potentially represent a compensatory mechanism and would then be consistent with insufficient tonic inhibition via GABA_A_Rs.

### Spine density and dendritic length

**Fig. 5.**
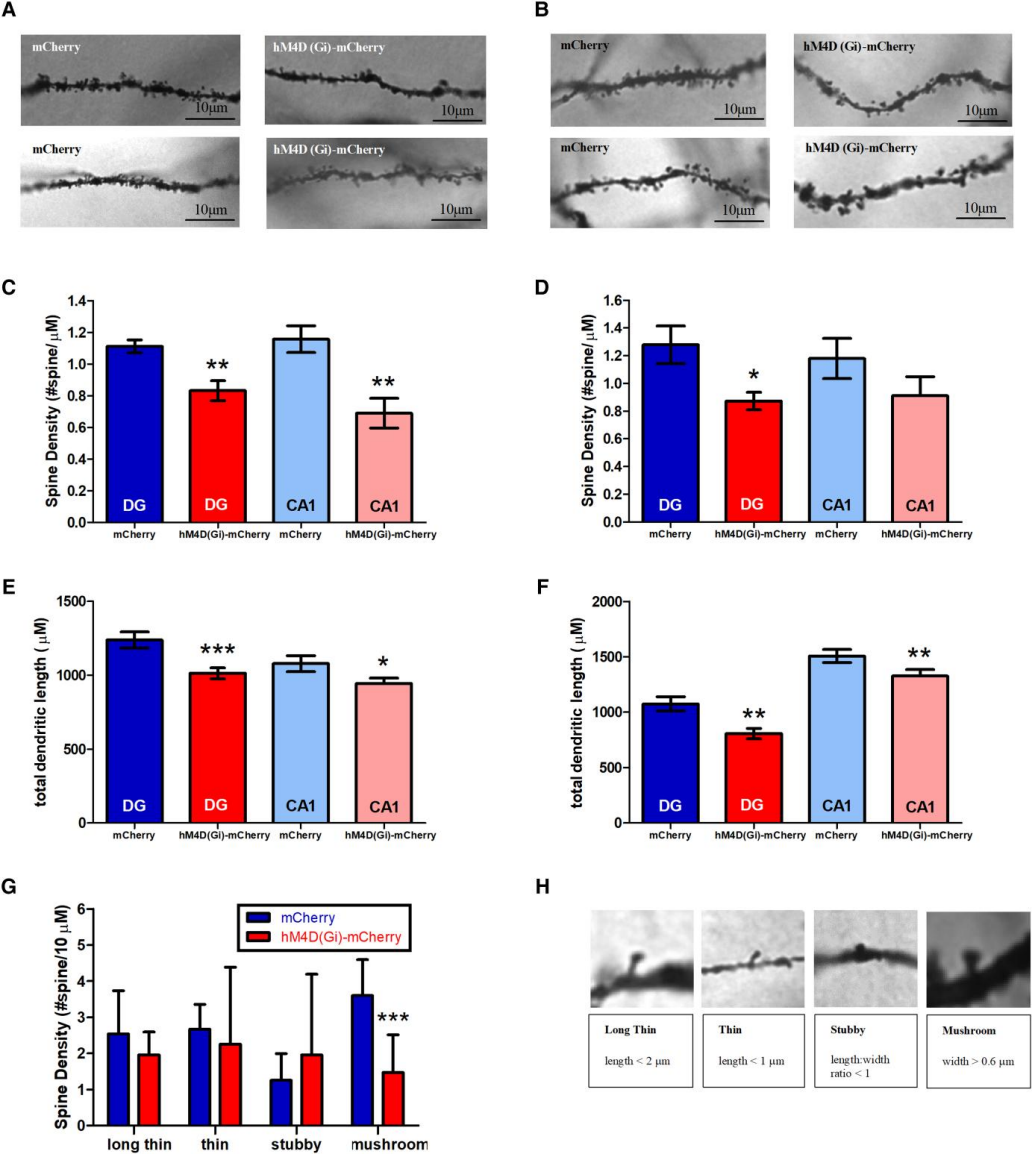
Morphological features of DG and CA1 regions in the hippocampus after CCI and SCI. A) Top: representative pictures of DG dendrite and spine morphologies after CCI used for quantification with a 100× objective. Bottom: representative pictures of CA1 dendrite and spine morphologies after CCI used for quantification with a 100× objective. B) Top: representative pictures of DG dendrite and spine morphologies after SCI used for quantification with a 100× objective. Bottom: representative pictures of CA1 dendrite and spine morphologies after SCI used for quantification with a 100× objective. C) Spine density in DG and CA1 after CCI. D) Spine density in DG and CA1 after SCI. E) Total dendritic length in DG and CA1 after CCI. F) Total dendritic length in DG and CA1 after SCI. G) Density of different spine types after CCI in DG. H) Top: examples of long thin, thin, stubby, and mushroom spines in DG after CCI. Bottom: quantification classification of different morphological spine types. *P < 0.05, **P < 0.01, and ***P < 0.001 compared with the corresponding mCherry control group.

To evaluate the impact of chronic and subchronic chemogenic inhibition of DG hilar Sst^+^ interneurons on hippocampal spine densities and spine length, we analyzed dendrites of granule cells located in the DG and apical dendrites of CA1 pyramidal neurons (Fig. 5A and B). Four mouse brains were collected for Golgi–Cox staining from each treatment group. Quantification of dendritic length and spine density in DG and CA1 region was performed on five to six neurons per mouse using *Reconstruct* software.

A two-tailed *t* test with Welch's correction of spine densities identified significant differences in DG between the AAV-hSyn-DIO-mCherry group and the AAV-hSyn-DIO-hM4D(Gi)-mCherry group (Fig. 5C, CCI: *t* = 3.757, *P* = 0.0027; Fig. 5D, SCI: *t* = 2.704, *P* = 0.0192), suggesting a decrease in spine density with chemogenetic inhibition of DG hilar Sst^+^ interneurons. A significant decrease was observed in the hM4D(Gi)-mCherry group compared with the mCherry group in the apical CA1 region only after CCI (*t* = 3.698, *P* = 0.0027), but not after SCI (*t* = 1.345, *P* = 0.1962) (Fig. 5C and D). However, total dendritic length was significantly reduced after SCI and CCI (Fig. 5E, DG in CCI: *t* = 3.435, *P* = 0.009; CA1 in CCI: *t* = 2.065, *P* = 0.0410; Fig. 5F, DG in SCI: *t* = 3.328, *P* = 0.0014; CA1 in SCI: *t* = 2.166, *P* = 0.0327), indicating a reduction in total dendritic length in DG and apical CA1 after chronic and subchronic inhibition of DG hilar Sst^+^ interneurons.

Dendritic spines are often categorized by their various morphological subpopulations, such as stubby, mushroom, thin, and long thin (Fig. 5H). We found a significant reduction of mushroom spines with other morphological types of spines unchanged (Fig. 5G; two-way ANOVA, group × spine type, *F*_(3,40)_ = 2.25, Diff = −2.126, *t* = 2.737, *P* < 0.05). CCI resulted in a reduced number of mushroom spines, which are associated with long-term memory storage (22).

### Cognitive function

Cognitive function has previously been reported to decline with aging in mice (23). To examine the impact of reduced activity of Sst^+^ interneurons on cognition, we performed the novel object recognition (NOR) task, the Morris water maze (MWM) test including reversal learning (RL), and the Y-maze (YM) task in both SCI and CCI experiments (Fig. 1B). It has been demonstrated before that performance in the NOR and the MWM is linked to CA1 (24–27), whereas RL is linked to the DG (14).

**Fig. 6.**
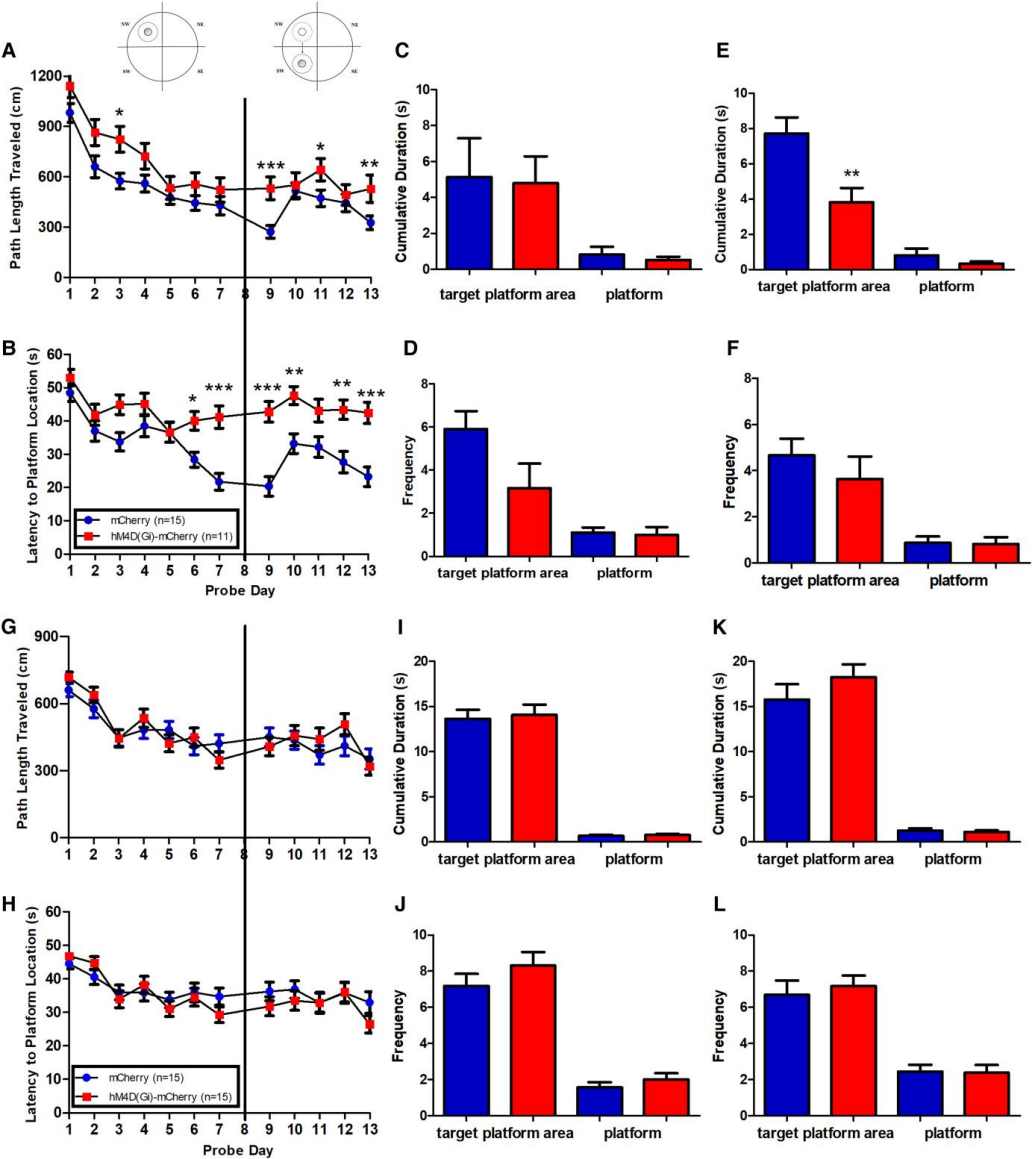
MWM in hM4D(Gi)-mCherry mice and mCherry mice. Days 1–7, L phase; day 8, L phase probe trials; days 9–13, RL phase; day 14, RL probe trials. A–F) CCI. A, B) Path length and latency to the hidden platform. C, D) Cumulative duration and frequency to enter target platform area or the platform in L phase probe trials. E, F) Cumulative duration and frequency to enter target platform area or the platform in RL phase probe trials. G–L) SCI. G, H) Path length and latency to hidden platform. I, J) Cumulative duration and frequency to enter target platform area or the platform in L phase probe trials. K, L) Cumulative duration and frequency to enter target platform area or the platform in RL phase probe trials. *P < 0.05, **P < 0.01, and ***P < 0.001.

To investigate the potential spatial memory deficits in the experimental mice, we performed the MWM (CCI: *n* = 11–15; SCI: *n* = 15; Fig. 6). A two-way mixed ANOVA followed by Bonferroni post hoc test was conducted to investigate the impact of “genotype” (AAV-hM4D(Gi)-mCherry vs. AAV-mCherry) and “day” on two behavioral measures, path length and latency, to find the hidden platform during learning (L) and RL phases. With CCI (Fig. 6A–F), the AAV-hM4D(Gi)-mCherry mice showed significantly increased path length (L: day, day × genotype, *F*_(5,76)_ = 1.547, *P* < 0.01; *t*_(7)_ = 2.773, *P* < 0.05 for day 3; RL: day, day × genotype, *F*_(4,76)_ = 1.575, *P* < 0.01; *t*_(5)_ = 4.540, *P* < 0.001 for day 9; *t*_(5)_ = 2.837, *P* < 0.05 for day 11; and *t*_(5)_ = 3.547, *P* < 0.01 for day 13) (Fig. 6A) and longer latency to find the hidden platform (L: day × genotype, *F*_(5,76)_ = 1.591, *P* < 0.01; *t*_(7)_ = 2.813, *P* < 0.05 for day 6; *t*_(7)_ = 4.70, *P* < 0.001 for day 7; RL: day × genotype, *F*_(4,76)_ = 2.634, *P* < 0.0001; *t*_(5)_ > 2, *P* < 0.01 for days 9, 10, 12, and 13) (Fig. 6B). With SCI (Fig. 6G–L), the AAV-hM4D(Gi)-mCherry mice did not differ from AAV-mCherry mice in their performance; no significant difference in latency or path length to find the hidden platform was observed between AAV-hSyn-DIO-mCherry and AAV-hSyn-DIO-hM4D(Gi)-mCherry mice (path length: *F*_(1,94)_ = 0.8596, *P* > 0.05, Fig. 6G; latency to find the hidden platform: *F*_(1,94)_ = 1.082, *P* > 0.05, Fig. 6H).

After the initial L phase and after the RL phase, we conducted probe trials to determine memory retrieval ability in experimental mice. In the CCI L probe trial, the AAV-hM4D(Gi)-mCherry mice displayed no significant difference for cumulative duration spent in the target platform area (unpaired *t* test, *P* > 0.05, Fig. 6C) or frequency to enter the target platform area (unpaired *t* test, *P* > 0.05, Fig. 6D). In the CCI RL probe trial, the AAV-hM4D(Gi)-mCherry mice spent a significantly shorter time than the AAV-mCherry mice in the target quadrant (Fig. 6E) (unpaired *t* test, AAV-hM4D(Gi)-mCherry: 7.708 ± 0.9183, *n* = 11; AAV-mCherry: 3.825 ± 0.8063, *n* = 15; *F*_(10,14)_ = 1.769, *P* = 0.3680). The frequency to enter the target quadrant was not different (unpaired *t* test, *P* > 0.05, Fig. 6F).

In the SCI L probe trial, AAV-hSyn-DIO-hM4D(Gi)-mCherry mice and AAV-hSyn-DIO-mCherry mice displayed no significant difference for cumulative duration in the target platform area and for the frequency to enter the target platform area (unpaired *t* test, *P* > 0.05, Fig. 6I and J). In the SCI RL probe trial, these two groups of mice displayed no significant difference for cumulative duration and frequency to enter the target platform area (unpaired *t* test, *P* > 0.05, Fig. 6K and L). The performance in the MWM L and RL phases revealed that the experimental mice had impaired performance only with CCI, while the SCI-treated experimental mice showed an intact performance with no significant differences found in either path length or latency to find the hidden platform during L, RL, and probe trials.

**Fig. 7.**
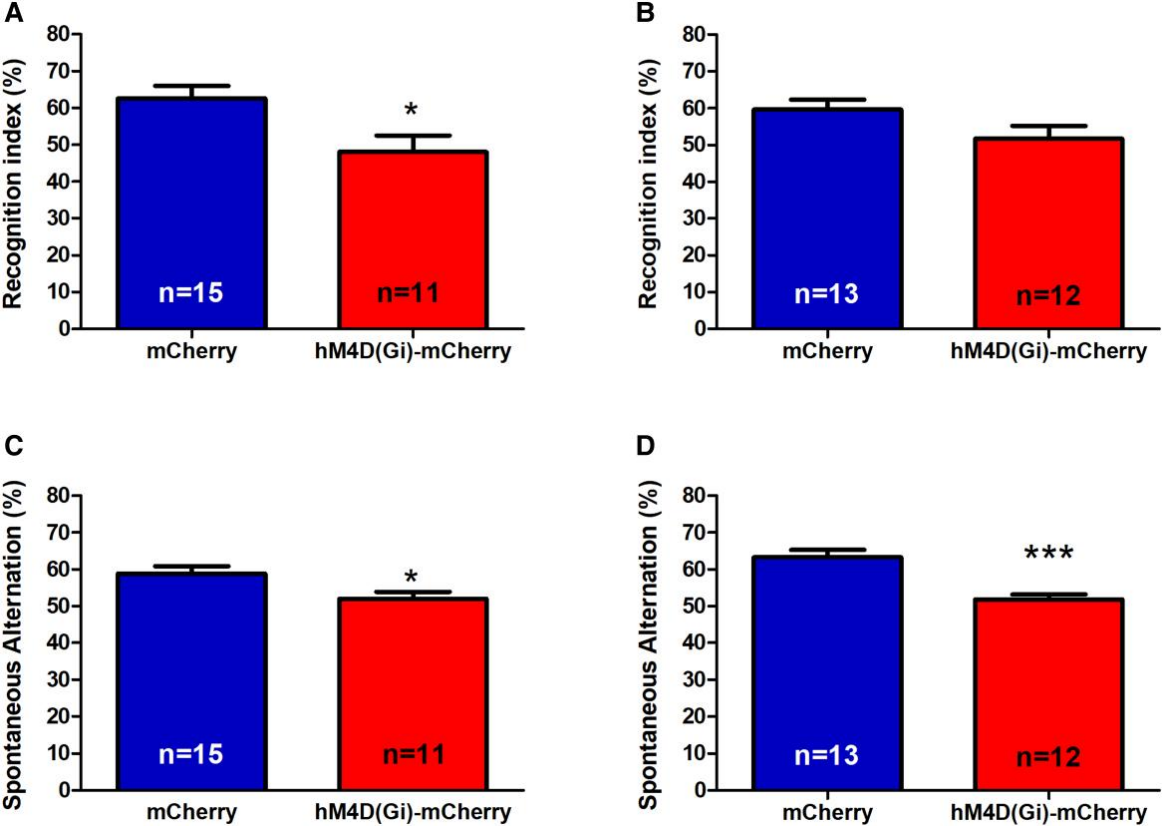
NOR task and YM task in AAV-hSyn-DIO-hM4D(Gi)-mCherry mice and AAV-hSyn_DIO-mCherry mice. A) Recognition index in NOR with CCI. B) Recognition index in NOR with SCI. C) Spontaneous arm alternation percentage in YM with CCI. D) Spontaneous arm alternation percentage in YM with SCI. *P < 0.05 and ***P < 0.001.

We then determined whether a reduced density of Sst^+^ interneurons in the DG hilus is associated with a decline in short-term recognition memory by analyzing AAV-hSyn_DIO-hM4D (Gi)-mCherry and AAV-hSyn-DIO-mCherry mice after both CCI and SCI in the NOR test. A significant decrease in the recognition index was observed with CCI (AAV-hSyn-DIO-hM4D(Gi)-mCherry: 62.65 ± 3.406, *n* = 15; AAV-hSyn-DIO-mCherry: 48.12 ± 4.352, *n* = 11; *P* = 0.0125, *t* = 2.670, df = 28, unpaired *t* test, Fig. 7A). In contrast, with SCI, both types of mice spent a similar amount of time in the proximity of the novel object (AAV-hSyn-DIO-hM4D(Gi)-mCherry: 51.77 ± 3.489, *n* = 13; AAV-hSyn-DIO-mCherry: 59.63 ± 2.752, *n* = 12; *P* > 0.05, unpaired *t* test, Fig. 7B), suggesting a decreased recognition memory in AAV-hSyn-DIO-hM4D(Gi)-mCherry mice with CCI but not with SCI.

The YM task was used to assess short-term working memory. With CCI condition, AAV-hSyn-DIO-hM4D(Gi)-mCherry mice had a significantly lower spontaneous alternation rate than their controls (AAV-hSyn-DIO-mCherry: 58.91 ± 2.005, *n* = 15; AAV-hSyn-DIO-hM4D(Gi)-mCherry: 52.06 ± 1.811, *n* = 11; *P* = 0.0102, *t* = 2.457, df = 28, unpaired *t* test) (Fig. 7C). With SCI, the spontaneous alternation rate also differed between groups with a lower recognition index in AAV-hSyn-DIO-hM4D(Gi)-mCherry mice (AAV-hSyn-DIO-mCherry: 63.31 ± 2.004, *n* = 13; AAV-hSyn-DIO-hM4D(Gi)-mCherry: 51.89 ± 1.362, *n* = 12; *P* < 0.0001, *t* = 4.806, df = 32; unpaired *t* test) (Fig. 7D). Thus, the AAV-hSyn-DIO-hM4D(Gi)-mCherry mice had a significantly lower YM spontaneous alternation rate than the AAV-hSyn-DIO-mCherry control mice in both SCI and CCI conditions.

## Discussion

Understanding the cellular mechanisms underlying aging is crucial to develop novel strategies to prevent or to reverse age-related cognitive dysfunction. Based on previous studies in aged rats, which showed that expression of both Sst and GAD-67 proteins in DG hilus interneurons is reduced with age (6) and that the reduction of DG hilar Sst^+^ interneurons is correlated with cognitive impairments (6, 28), we investigated two main questions: (i) whether a reduced activity of Sst^+^ interneurons in the DG hilus is sufficient to alter the activity of principal neurons in hippocampal subregions, to lead to changes in the expression of cellular markers and to impair of cognitive functions, and, if this is the case, (ii) whether chronic (vs. subchronic) inhibition is required for these effects. The subchronic inhibition protocol was intended to obtain an effect that is closer to acute inhibition, as truly acute inhibition is not feasible when using the same animals in multiple tests. While the daily dose of clozapine is the same in both protocols (0.1 mg/kg per day), given the half-life of clozapine in mice of 110.1 ± 8.1 min (29), the subchronic protocol with daily intraperitoneal injections likely creates once daily peaks, whereas the chronic protocol with administration of clozapine in the drinking water likely results in more constant, possibly steady-state–like clozapine levels. Overall, the results show that chronic inhibition is necessary for many of the phenotypes seen with CCI, and thus, chronic inhibition is necessary to get the full aging-related phenotype.

### CCI of Sst^+^ hilar interneurons

Our experiments with CCI of Sst^+^ interneurons in the DG hilus are summarized in Table 1. CCI resulted in an increase in the number of c-Fos–positive neurons in the DG, but not in CA3 or CA1 (Fig. 2), a reduction in the number of GAD67^+^ and Sst^+^ interneurons in the DG, but not in CA3 or CA1 (Fig. 3), and an increase in the Iba1^+^ cell body size (pixel) indicating microglial activation in DG, CA3, and CA1 (Fig. 4). The dendritic spine density was reduced in both DG and CA1 (Fig. 5). Behaviorally, L and RL in the MWM were impaired (Fig. 6), as were NOR and alternation in the YM (Fig. 7). The results clearly demonstrate that CCI of Sst^+^ interneurons in the DG hilus is sufficient to induce cellular changes and cognitive dysfunction.

**Table 1.** Expression of molecular markers, behavioral and morphological effects of CCI and SCI of DG hilus Sst^+^ interneurons.

	c-Fos	Sst	GAD67	Iba1	MWM	NOR	YM	DG spine density	DG dendritic length	CA1 spine density	CA1 dendritic length
CCI	↑	↓	↓	↑	↓	↓	↓	↓	↓	↓	↓
SCI	↑	−	−	−	−	−	↓	↓	↓	−	↓

The displayed results are comparing outcomes in AAV-hSyn-DIO-hM4D(Gi)-mCherry mice compared with AAV-hSyn-DIO-mCherry mice. Most molecular and behavioral changes require CCI.

### SCI of Sst^+^ hilar interneurons

When studying the effects of SCI of Sst^+^ interneurons in the DG hilus (summarized in Table 1), we found an increase in the number of c-Fos–positive neurons in the DG, but not in CA3 or CA1, similar to what we observed with CCI (Fig. 2). In contrast to CCI, SCI did not reduce the number of GAD67^+^ and Sst^+^ neurons (Fig. 3), and, also in contract to CCI, SCI did not increase Iba1^+^ cell body size (pixel) in DG, CA3, and CA1, i.e. apparently did not induce microglial activation (Fig. 4). SCI leads to a reduction of dendritic spine density in DG (as with CCI) but not in CA1 (unlike CCI) and to a reduction of total dendritic length in both DG and CA1 (as with CCI) (Fig. 5). Behaviorally, unlike CCI, SCI had no effect on L and RL in the MWM (Fig. 6) and in the NOR test (Fig. 6), but like CCI, SCI reduced the spontaneous alternations in the YM (Fig. 7).

### Comparison of CCI and SCI

Our studies revealed that while both SCI and CCI result in activation of neurons in the DG, a reduction in the number of GAD67^+^ and Sst^+^ interneurons in the DG is only observed with CCI, not with SCI. Likewise, microglial activation in DG, CA3, and CA1 was only observed with CCI and not with SCI. Finally, spatial memory and NOR memory were impaired only with CCI, but not with SCI. Only when testing working memory in the YM, both SCI and CCI reduced the percentage of correct alternations, and both SCI and CCI led to reductions in spine density and dendritic length. One potential interpretation for this result is that while chronic inhibition is required to develop the full phenotype observed after CCI, the SCI protocol also has a “chronic” component. Notably, the experiments in which SCI induced some of the changes seen with CCI were performed after more than two weeks of once daily treatment with clozapine: the YM test after 17 days and the cellular tests after 18 days. In any case, the cellular data show some clear differences between SCI and CCI, indicating that these treatment protocols were substantially different. Moreover, the clear differences in some assays demonstrate that chronic inactivation of Sst^+^ interneurons is required for key elements of the observed phenotype with CCI. In our model, chronic inhibition of ∼63% of the hilar Sst^+^ interneurons results in cognitive dysfunction, which is in line with the observation that in aged cognitively impaired rats, the number of the hilar Sst^+^ neurons is reduced by approximately half (6). Overall, these results strongly support the hypothesis that chronic inhibition of Sst^+^ interneurons in the DG hilus is necessary and sufficient for microglial activation and cognitive dysfunction. This dependence on chronic inhibition of these neurons for inducing cognitive dysfunction also aligns with the chronicity of the aging process, supporting the view that a gradual decline of function of hilar Sst^+^ interneurons, as likely occurs with age-related reduction in the number of hilar Sst^+^ interneurons (6, 28), contributes to age-related cognitive decline. An alternative explanation could be that in aging, either chronic psychosocial stress leading to an exacerbated unfolded protein response of the endoplasmic reticulum or reduced proteostasis capacity (a hallmark of aging) results in Sst protein aggregation negatively affecting the function of Sst^+^ interneurons, as observed in the prefrontal cortex (30). When considering CCI as a potential model for hippocampal aging, our results are in line with previous studies which showed memory impairments in MWM, YM, and NOR in aged rodents (31–33), indicating that memory impairment and aging are closely linked and may be dependent on the same hippocampal neural circuits (14, 25, 34).

While the current study was underway, Fee et al. reported a study on the role of Sst^+^ cells in regulating mood and cognitive functions (35). They used a global approach and developed a chemogenetic model which inhibits Sst^+^ interneuron function brain-wide by injecting their chemogenetic inhibitory receptor-containing viral construct intraventricularly. They employed clozapine *N*-oxide (CNO) to activate the *d*esigner *r*eceptor *e*xclusively *a*ctivated by *d*esigner *d*rugs (DREADD) instead of clozapine used in the current study and also performed the YM and NOR tasks. They achieved repeated acute silencing of Sst^+^ cells by administrating CNO 30 min before each behavioral experiment, which is similar to our SCI model. While they found that their CNO-treated mice spent significantly less time with the novel object than with the familiar object in the NOR task and there were no differences between groups in the YM task, with subchronic injections of clozapine, we found no difference between AAV-hSyn-DIO-mCherry-injected mice and AAV-hSyn-DIO-hM4D(Gi)-mCherry-injected mice in the NOR task, but AAV-hSyn-DIO-hM4D(Gi)-mCherry-injected mice displayed significantly fewer spontaneous alternations in the YM task. Although the reasons for the observed differences are not understood, there are some potential explanations, including differences in dosing (3.5 mg/kg CNO per day in Fee et al. vs. 0.1 mg/kg clozapine per day in our SCI and CCI studies) (35), multiple experiments being performed with repeated acute dosing which might result in chronic-like effects especially at higher drug doses, and their generalized Sst^+^ interneuron inhibition that could affect multiple neural circuits, while we inhibited a more narrowly targeted cell population. In any case, both studies demonstrated effects of Sst^+^ cell regulation on cognitive functions. Interestingly, a difference between acute and chronic chemogenetic silencing of Sst^+^ interneurons has also been reported by Soumier et al. (36), who described that while acute chemogenetic inhibition of Sst^+^ interneurons in the frontal cortex resulted in increased behavioral emotionality, CCI resulted in decreased behavioral emotionality.

Several lines of evidence point to a role of GABAergic inhibition for hippocampal learning and memory. In mice in which hilar GABAergic interneurons were silenced optogenetically, c-Fos^+^ expression in the DG (but not in CA3 or CA1) and the firing rate of the GCs were increased, resulting in impaired spatial learning and memory retrieval (7). Aged rats with memory impairment had restoration of hilar Sst expression after receiving levetiracetam, a compound that modulates synaptic neurotransmitter release (6). Mice with decreased GABA levels in the hippocampus induced by overexpressing GABA transporter 1, which mediates GABA reuptake, display deficits in learning and memory (37). A lack of α5-GABA_A_Rs in the DG granule cells, which presumably led to a reduced inhibitory input into the granule cells, has previously been shown to result in a reduced tonic inhibition of DG granule cells and RL deficits in the MWM (14). All of these studies thus revealed a critical role of GABAergic neurotransmission for hippocampal learning and memory. Thus, our current study demonstrating that inhibition and thus reduced activity of a GABAergic interneuronal subtype result in cognitive deficits is line with previous evidence demonstrating essential roles for GABA in learning and memory. Moreover, GABA levels in the mouse brain as determined by ^1^[H] magnetic resonance spectroscopy are reduced with aging (38). Moreover, increased microglial activation is known to be associated with human aging (39). In our study, we found microglial activation in DG, CA3, and CA1 regions in CCI mice, indicating the inactivation of Sst^+^ interneurons causes changes in the hippocampus that eventually result in an increase of this microglial activation marker.

### Conclusion and future directions

In summary, our experiments demonstrate a causal relationship between a chronic loss of activity of dentate hilus Sst^+^ interneurons, microglial activation, and cognitive dysfunction. Given that a reduction in the number of these interneurons has been found to be associated with age-related memory deficits (6, 28), a reduced number or function of hilar Sst^+^ interneurons is thus likely to be an important factor in the development of age-related cognitive dysfunction. Unlike the SCI model which also results in increased c-Fos staining and hyperactivity in DG, the CCI model mimics several relevant features of hippocampal aging and may thus be further evaluated as an experimental model of aging-related processes in the hippocampus, which also allows to study the potential reversibility of the phenotype after stopping clozapine administration. This novel model may also be useful to study postoperative neurocognitive disorder and other age-related cognitive disorders and for the development of novel therapeutic approaches for such disorders.

## Materials and methods

### Animals

Adult Sst-IRES-Cre (Sst-Cre) transgenic mice (Stock no. 013044, The Jackson Laboratory) were crossed with C57BL/6J mice (Stock no. 000664, The Jackson Laboratory). Hemizygous adult mice of both sexes (3–4 months) were used for all studies. All mice were housed in a climate-controlled room maintained on a 12:12 light–dark cycle (lights on at 7 AM and lights off at 7 PM) with food and water ad libitum. All procedures are approved by the Institutional Animal Care and Use Committee at University of Illinois at Urbana-Champaign. ARRIVE guidelines were followed.

### Silencing Sst^+^ cell function in the DG hilus

To achieve a Sst^+^ cell-specific manipulation, we used Sst-Cre mice, which express Cre recombinase in Sst-expressing neurons. We bred heterozygous Sst-Cre mice with wild-type mice to generate experimental Sst-Cre mice. A G_i_-coupled DREADD was used that decreases the overall amount of cAMP, which results in neural inhibition (40, 41). To achieve region-selective manipulation, we stereotaxically injected a viral G_i_-DREADD construct that will only be expressed in Cre-positive cells into the DG hilus region, where it can be activated with CNO or clozapine. While CNO is likely the most widely used ligand for chemogenetic studies, clozapine displays higher affinity and greater potency for hM4D(Gi) (41). Clozapine has favorable pharmacokinetic properties for chronic administration (42, 43) and was therefore used in our experiments.

### Surgery

In order to study the role of Sst^+^ in the dentate hilus, we generated mice in which the Sst^+^ interneurons in the dentate hilus can be silenced chemogenetically. Sst-Cre mice (3–4 months of age) received ketoprofen 5 mg/kg, s.c., and atropine 0.04 mg/kg, s.c., before surgery. Mice were anesthetized with isoflurane (2–3%) and maintained under anesthesia (1.5%) throughout the surgery. AAV-hSyn-DIO-hM4D(Gi)-mCherry (Addgene #44362) was injected bilaterally into the DG hilus (stereotaxic coordinates AP: 2.1 mm, ML: ± 1.5 mm, and DV: −2.1 mm relative to bregma) at a rate of 0.120 µL/min (1,000 nL per side), and the needle was left in place for an additional 2 min to permit diffusion. The control groups were injected with an AAV vector, AAV-hSyn-DIO-mCherry (Addgene #50459), lacking the chemogenetic receptor hM4D(Gi). Injection locations were verified histologically at the end of the study in 35 mice (while 16 mice, 8 from CCI and 8 from SCI experiments, were used for Golgi staining). Of 35 mice examined for mCherry immunofluorescence, the DG hilus was hit bilaterally in 32 mice. One mouse in the CCI group (injected with the AAV-hSyn-DIO-hM4D(Gi)-mCherry construct) and two mice in the SCI group (injected with the AAV-hSyn-DIO-mCherry construct) had unilateral hits. Animals in which both injection sites were missed were removed from the study.

Clozapine dihydrochloride (water soluble, Hello Bio #HB6129) was administered to the mice to activate hM4D(Gi) and thus inhibit hilar Sst^+^ interneurons. The chronic treatment groups received clozapine (0.1 mg/kg/day) in the drinking water for 21 days before and throughout all testing and the subchronic treatment groups were administered clozapine (0.1 mg/kg i.p.) 30 min before behavioral experiments started. Mice drink ∼4 mL of water per day. Males weigh more than females (e.g. 35 g vs. 25 g, so the males may get 3.5 μg/4 mL and the females 2.5 μg/4 mL). Mice were weighed every 2 days, and the clozapine solution adjustments made every 2 days to reach the desired concentration.

### Behavioral tests

All behavioral experiments were performed during the light phase of the light/dark cycle. All mouse behaviors were monitored using the EthoVision XT video tracking system.

#### NOR task

Mice were habituated in the experimental chamber for 2 days before the testing phase and each habituation period lasted for 15 min for each animal per session. Each mouse was presented with two identical objects for 10 min on the actual test day. After 1 h, the mice were brought back to the same chamber and presented with one of the same training objects and one novel object. Interaction time was recorded using the multiple body point module. Mice were considered exploring the novel object when the nose point as defined by the EthoVision XT software was in close proximity to the object. A novelty recognition index was calculated by dividing the time spent in the proximity of the novel object by the total time spent with both objects.

#### MWM L and RL

A round pool (diameter: 120 cm) is filled with a mixture of water (22–24°C) and a white, nontoxic dye (Blick Premium Grade Tempera). A 10-cm-diameter platform was submerged 2 cm under the water surface. A 25-cm-diameter target platform area was defined in the same quadrant as a concentric circle around the target platform. The frequency to enter and cumulative duration spent in the target platform area are also recorded and analyzed. Visual cues were in the four quadrants of the pool in shapes with different geometry. Mice performed three trials daily from day 1 to day 7, released from a different quadrant each time in random order while the target platform location was constant. A trial ended when the animal found the platform and stayed on it for 2 s. When an animal failed to reach the platform within 60 s, the experimenter guided it to the platform and put it back to the cage after staying on the platform for 10 s. From day 9 to day 13, the RL phase was established by moving the platform from the original location to the nearest quadrant to increase the effects of interference. During probe trial (day 8) and RL probe trial (day 14), the platform was removed, and the mice were left in the pool for 120 s.

#### YM task

The apparatus was a Y-shaped maze with three gray, opaque plastic arms at a 120° angle from each other. The arm length is 31 cm and the arm width is 7 cm. Mice were placed in the center of the YM and allowed to explore any of the three closed arms freely for 8 min. The spontaneous arm alternation rate is calculated by dividing the number of spontaneous alternations by the number of total arm entries and multiplication by 100. Mice with intact working memory would run in the three arms with spontaneous alternations, meaning that at each left–right decision point, they make an alternating decision. In the YM, a spontaneous alternation rate of 50% would imply random choices.

### Histology and immunohistochemistry

Mice were deeply anesthetized with ketamine (139 mg/kg i.p.) and xylazine (21 mg/kg i.p.) and then transcardially perfused with ice-cold phosphate-buffered saline (PBS) followed by 4% ice-cold paraformaldehyde. After 24 h of postfixation at 4°C, brains were moved into 30% sucrose for 72 h and sectioned into 30-µm coronal sections using a cryostat. Free-floating sections were collected mounted on slides for staining.

We performed analysis of sections for target cell labeling and quantification. For analysis of c-Fos staining, all mice (*n* = 6 per group) were exposed to a novel environment for 10 min and then placed into a clean cage individually for 1 h before perfusion. For other stainings, free-floating sections (*n* = 3 per sex, *n* = 6 per group) were washed in 0.1 m PBS to remove cryoprotectant. The sections were blocked in blocking solution (2% NGS and 0.4% Triton X-100 in PBS) for 2 h at room temperature, then incubated in the primary antibody at 4°C overnight. The primary antibodies used were as follows: rabbit anti-c-Fos (Cell Signaling Tech, cat # 2250, 1:500), anti-Sst (SOM, Invitrogen, cat # PA5-82678, 1:1,000), antiglutamic acid decarboxylase 67 (GAD-67, Invitrogen, cat # PA5-21397, 1:500), anti-Iba1 on microglia (Iba1, Invitrogen, cat # MA5-36257, 1:500), and antineuronal nuclei (NeuN, Cell Signaling Tech, cat # 24307, 1:400). After washing in blocking solution and 3% H_2_O_2_, the sections were incubated in a biotinylated goat antirabbit secondary antibody in blocking solution (Invitrogen, cat # 31820, 1:500) and transferred in a detection reagent (Vecstatin Elite ABC kit, Vector Laboratories, cat # PK-7200). The sections were incubated in a 3,3′-diaminobenzidine solution. After rinsing, sections were mounted, dehydrated, and cover-slipped with mounting medium (Eukitt Quick-handling). The section slides were imaged with an Olympus BX51 microscope.

To verify viral placements and immunofluorescence labeling of mCherry, DAPI stain (Abcam, cat # ab228549) was added to free-floating 30-µm coronal sections using the concentration of 1 µm in the dark. The sections were mounted and cover-slipped on slides using Eukitt Quick-handling mounting medium and imaged with a Leica DM2500 microscope. Each slide was checked and validated for bilateral hits or at least unilateral hits, with approximately six to seven sections covering the dorsoventral axis of the DG.

To investigate the transduction efficiency of our constructs, AAV-hSyn-DIO-mCherry virus was injected into the DG hilus of Sst-Cre mice (*n* = 4). Three weeks later, animals were perfused, and brain slices were colabeled with anti-Sst primary antibody (SOM, Invitrogen, cat # PA5-82678, 1:100) and an Alexa Fluor 488 dye-labled goat antirabbit secondary antibody (Thermofisher, cat #A-11008, 1:500), then counterstained with DAPI (Abcam, cat # ab228549, 1:500).

### Golgi–Cox staining

Mouse brains were removed from skulls without fixation. The fresh brain tissue was immersed in the impregnation solution (superGolgi Kit, Bioenno Tech) for 12 days and stored at 4°C in the dark. After being transferred to and incubated in the postimpregnation buffer for 2 days, the brain tissues were sectioned into 150-µm-thick sections using a vibratome. The sections were collected and mounted on gelatin-coated slides, dehydrated, and cleaned. Then they were cover-slipped using a mounting medium (Sigma-Aldrich) and stored at room temperature in the dark.

### Dendritic spine analysis

We examined spines on apical dendrites of CA1 pyramidal neurons and dendrites of the dorsal DG. To ensure the accurate measurements, we only evaluated dendrites that showed no breaks in the staining (44) and that were not interrupted by other neurons or artifacts (45). Primary spines were not analyzed; we only evaluated spines located on secondary or tertiary dendritic trees. One segment per individual dendritic branch and two branches per neuron were chosen for the analysis. Quantitative 2D analyses of dendritic and spine fragments were conducted by using a stereological microscope (Zeiss AxioImager A1 light microscope) with a 100× objective (oil immersion). For each dendrite, at least three images along each spine segment were taken and spine densities calculated. Spine densities per 10 µm and spine length from the dendrite shaft to the spine head were marked and calculated by using *Reconstruct* software (Version 1.1.0.1) (46).

### Iba1 quantification in microglia

Iba1 staining was analyzed using ImageJ (ImageJ 1.53v). The Iba1^+^ pixel density was determined by using thresholds such that only pixels within a range of color intensities and cell sizes were included for analysis. The particle intensity threshold was adjusted based on the background of the image to exclude nonspecific signal. ImageJ calculated cell body size in the unit of “pixel.”

### Statistical analysis

Statistical comparisons were performed using Graph Pad Prism (Graph Pad Software Inc., La Jolla, CA). In the c-Fos, SOM, GAD67, and NeuN staining experiments, the average numbers of c-Fos^+^ nuclei, Sst^+^, GAD67^+^ cells, and NeuN^+^ cells in the DG, CA3, and CA1 regions were analyzed using two-way repeated measures ANOVA. Each slide was counted three times, and the mean value was used in the ANOVAs. The average Iba1^+^ cell body size was analyzed with one-way ANOVA followed by Newman–Keuls multiple comparison tests. Statistical differences between treatments and regions were assessed using Bonferroni's post hoc comparison test. Performance in behavioral experiments was analyzed using one-way ANOVA and Scheffe's test.

## Data Availability

All data are included in the manuscript. Primary data have been deposited in the Harvard Dataverse: https://doi.org/10.7910/DVN/OFITGT.
